# Searching for motifs in the behaviour of larval *Drosophila melanogaster* and *Caenorhabditis elegans* reveals continuity between behavioural states

**DOI:** 10.1098/rsif.2015.0899

**Published:** 2015-12-06

**Authors:** Balázs Szigeti, Ajinkya Deogade, Barbara Webb

**Affiliations:** 1 Neuroinformatics Doctoral Training Centre, University of Edinburgh, Edinburgh, UK; 2 EMBL-CRG Systems Biology Program, Barcelona, Spain; 3 School of Informatics, University of Edinburgh, Edinburgh, UK

**Keywords:** *Drosophila melanogaster* larva, *Caenorhabditis elegans*, eigenworms, eigenmaggots, behavioural states, ethogram

## Abstract

We present a novel method for the unsupervised discovery of behavioural motifs in larval *Drosophila melanogaster* and *Caenorhabditis elegans*. A motif is defined as a particular sequence of postures that recurs frequently. The animal's changing posture is represented by an eigenshape time series, and we look for motifs in this time series. To find motifs, the eigenshape time series is segmented, and the segments clustered using spline regression. Unlike previous approaches, our method can classify sequences of unequal duration as the same motif. The behavioural motifs are used as the basis of a probabilistic behavioural annotator, the eigenshape annotator (ESA). Probabilistic annotation avoids rigid threshold values and allows classification uncertainty to be quantified. We apply eigenshape annotation to both larval *Drosophila* and *C. elegans* and produce a good match to hand annotation of behavioural states. However, we find many behavioural events cannot be unambiguously classified. By comparing the results with ESA of an artificial agent's behaviour, we argue that the ambiguity is due to greater continuity between behavioural states than is generally assumed for these organisms.

## 1. Introduction

Automated analysis of behaviour is of increasing importance to biology and neuroscience. Behavioural control is the ultimate function of neural processing [1]. The recent expansion of tools for manipulating neural activity, such as optogenetics, has made it crucial to be able to screen rapidly and automatically for the behavioural consequences of these manipulations. Standardization of quantitative behavioural assays and reproducibility of analyses are thus key to progress in understanding neural circuits.

Traditional manual annotation of behavioural data is not feasible for large datasets. As a consequence, automated high-throughput behavioural annotators have been developed. An example is the Janelia Automatic Animal Behaviour Annotator (JAABA) [2]. JAABA first requires hand annotation of a subset of the data and then the software uses machine learning algorithms to find the same patterns in the unannotated data. Other researchers have developed classifiers that extract specific parameters from behavioural data and then register a state if a certain parameter (or parameter set) exceeds a user-defined threshold [3–6]. Note that for these classifiers both the set of possible behaviours and the description of those behaviours are encoded by the user. In contrast, our goal is to discover patterns in behaviour without reference to any user-defined thresholds or examples.

**Figure 1.**
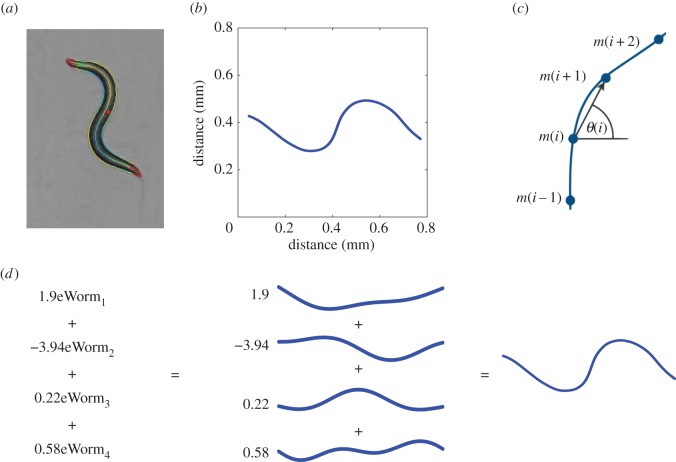
Constructing eigenworms. In each video frame, thresholding is used to separate the animal from the background, then the resulting binary images are skeletonized. This skeleton, or midline, is used as a proxy for the animal's posture. Panel (*a*) shows a frame from the CBD with the worm's contour and midline highlighted, panel (*b*) shows the corresponding midline. The skeleton has been rotated to remove the worm's overall rotation relative to the plate. Panels (*c*) zooms in on the midline, showing how a set of *θ_i_* angles provide a piecewise linear approximation to the midline curvature. This angular data forms a vector for each frame, or a matrix for a movie. The matrix's principal components are the eigenworms. Panel (*d*) shows an example of a posture reconstruction. The blue shapes in the middle column are the eigenworms, which can be added together with different weights to reconstruct any actual worm posture.

Posture is the main observable component of behaviour, and the behavioural annotators mentioned above mainly use postural information as input to classify behavioural states. In this context, Stephens *et al*. introduced eigenworms [7], using principal component analysis to produce a low-dimensional representation of *C. elegans* midline shapes. For the unrestricted free behaviour of *C. elegans*, four eigenworms account for 92% of the animal's posture variance. This means that four numbers can describe any actual worm posture with high precision. Mathematically, postures are described by a superposition of eigenworms, i.e.1.1
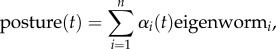
where *α_i_*(*t*) is the coefficient associated with the *i*th eigenworm at time *t*. Figure 1 shows the eigenworms and an example of posture reconstruction. Eigenshapes provide a compact representation of posture and hence clearly have potential use in behavioural annotation. Specifically, behaviour (change in posture over time) is represented by the time evolution of eigenshape coefficients, i.e. the time series of *α_i_*(*t*)s. This time series will be referred to as the eigenshape coefficient time series (ECTS) and forms the basis of our method.

The technical aim of this paper is the unsupervised discovery of frequently repeated ECTS subsequences. In the data mining literature, frequently repeated subsequences are also known as motifs [8]. ECTS motifs correspond to frequently repeated sequences of posture that can be viewed as behavioural states or actions [9,10]. Previous attempts to extract ECTS motifs using a simple ‘sliding window’ motif discovery approach [11] suffer from two major problems. First, the window for any pass is of fixed length, hence this method considers only exactly equal duration sequences as potential matches. Second, the sliding window method defines a motif as a *pair* of closest neighbour sequences. However, motifs are understood intuitively not as a single pair of subsequences, but as a *frequently repeated* subsequence. Our motif finding methodology was designed to overcome these two problems.

First, we derive the equivalent of eigenworms for larval *Drosophila*, termed eigenmaggots. The ECTS of both larval *Drosophila* and *C. elegans* are then analysed using our novel motif finding method. The ECTS motifs are used as the basis of a probabilistic behavioural annotator, the eigenshape annotator (ESA).^1^ We show that the resulting annotation corresponds well to hand annotation, although a number of behaviours cannot be unambiguously classified. The ESA analysis is also applied to the behaviour of a state-based simulated maggot to show that the ambiguity is not inherent in the method, but reflects a greater continuity between behavioural states in these organisms than is generally assumed. In summary, our new method both confirms the results of previous behavioural annotation and reveals some of its limitations.

## 2. Methods

### 2.1. Overview

Our aim is to go from video of a behaving animal to annotation of its behavioural states, where those states are determined using bottom-up discovery of motifs in the sequence of postures. We start by recording freely foraging *Drosophila* larva, extract their midline as a set of angles, and apply principal component analysis to obtain a low-dimensional description of postures, the ECTS. Equivalent information for the worm is available from the *C. elegans* behavioural database (CBD). Discovering motifs in the multidimensional ECTS is a non-trivial problem, and there are no existing adequate tools. We developed a two-step process to first extract subsequences and then fit a statistical model to cluster the subsequences. Briefly (details are given below), we use changes in the dynamics of the ECTS to divide the sequence into variable length subsequences, with the intent that each subsequence contains a single ‘action’. The subsequences are aligned and then clustered using a spline regression model [12,13], a method for analysing curves analogous to Gaussian mixture models. The resulting clusters constitute motifs by which the animal's behaviour can be annotated. The results are compared with alternative annotation systems and with hand annotation provided by a human expert, which is treated as ground truth.

### 2.2. Data collection

Canton-S flies were maintained on conventional cornmeal-agar molasses medium at 22°C and kept in a 12 h dark–light cycle. For the behavioural experiments, larvae in their 3rd instar stage were placed on 3% agarose and were allowed to freely forage. Across 33 individuals, 14 h of video was recorded at 30 fps. The videos were segmented (see below) into a total of 11 613 actions. The tracking and data acquisition hardware used for this publication are described in detail in [14]. Briefly, the larva moving over a fixed stage was imaged using a camera (Basler A622f) on top. The camera was mounted on a moving stage to follow the animal. The software for image capture and stage control was written in C using the OpenCV libraries.

To analyse worm behaviour, we used data from the *CBD* [6]. The database consists of videos of worms (recorded at 30 fps) browsing in bacteria. For every video, there is a corresponding feature file, which contains many precalculated statistics of worm morphology. The feature files also contain the eigenworm coefficient time series. The worm analysis in this paper uses this precalculated ECTS. Twenty-two thousand and sixty-six actions were analysed from 100 experiments with N2 worms, corresponding to 25 h of video.

### 2.3. Constructing eigenmaggots

**Figure 2.**
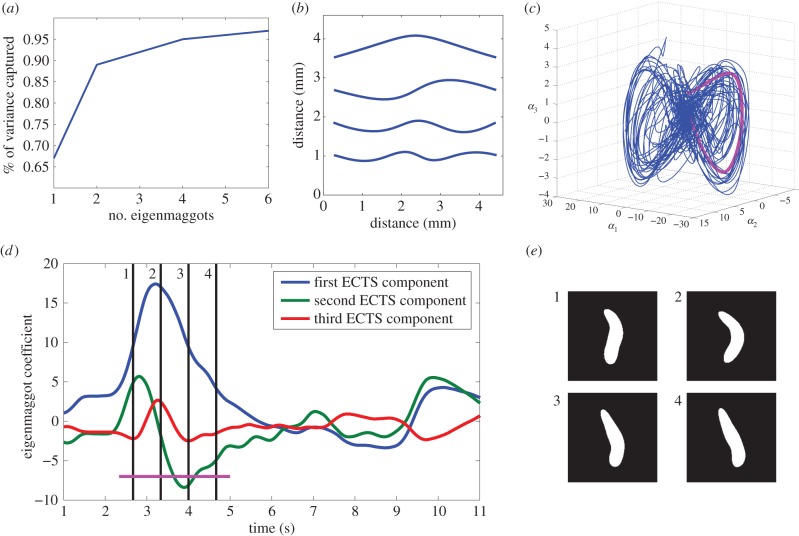
Results of eigenmaggot analysis. Panel (*a*) shows the percentage of the original data's variance recovered given the dimensionality of the representation. Panel (*b*) shows the eigenmaggots with the most significant eigenmaggot on top, the second below, etc. These shapes can be added up in different proportions to reproduce the larval postures (figure 1). Panel (*c*) shows a three-dimensional behavioural trajectory in eigenmaggot space, that is the time evolution of the first three eigenmaggot coefficients. The subtrajectory highlighted is an example of what we call a *turning manoeuvre*, see §3.2. Panel (*d*) shows a part of the same trajectory as three separate one-dimensional time series; the subsequence underlined corresponds to the highlighted subtrajectory on panel (*c*). Panel (*e*) shows binary images of the maggot at the corresponding time slices from panel (*d*).

In each video frame, the larva was separated from the background by a thresholding algorithm. The resulting binary images were skeletonized using the built-in MATLAB function [15]. Midlines were rotated such that the endpoints, corresponding to the head and tail of the animal, lie along the *x*-axis. This operation removes the overall rotation of the animal's body relative to the plate. The midlines were normalized such that they consist of 71 points placed equidistant from each other. The length of the larva can change, but is neglected in this analysis, i.e. we treat every midline as if it is the same length. The eigenshapes in figures 1 and 2 have been reconstructed to reflect the average physical size of the midlines. The angles among consecutive points defining the midline were restricted to the interval –*π* *<* *θ_i_* ≤ *π*. As a result of these operations, each frame is associated with a 70 dimensional vector, where the *i*th component is *θ_i_* (figure 1*c*). These vectors are concatenated to form an *n* * 70 data matrix, where *n* is the number of frames. Principal component analysis is applied to this data matrix to construct the eigenshapes and the associated ECTS.

### 2.4. Eigenshape coefficient time series

For both the larval and worm analysis, the coefficients of the three most significant eigenshapes were included in the ECTS, that is 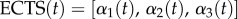, see equation (1.1). After principal component analysis, the inspection of the eigenvalues reveals that for both organisms three coefficients account for approximately 90% of the posture variance [16], thus provide an accurate description of posture. At the same time, a three-dimensional ECTS is small enough to avoid ‘the curse of dimensionality’ that could lead to difficulties during the clustering step [17].

### 2.5. Dropped frames

Both the larval and the maggot ECTS contains dropped frames. If a gap was short (less than 0.5 s), then ECTS was linearly interpolated. After the interpolation, 1.1% of the *Drosophila* and 4.2% of the *C. elegans* frames were still missing. For both organisms on a significant portion of the dropped frames, the animal was curled up in a ‘doughnut shape’ from which it is difficult to extract a biologically meaningful skeleton. For *C. elegans,* more frames were dropped, because the worms were browsing in food. The layer of bacteria can obscure the worm in the image, making separation of the body of the worm from the background more challenging. Note that the inability to analyse curled-up postures introduces a bias to the pipeline, as no posture with self-intersection is included.

### 2.6. Segmentation

**Figure 3.**
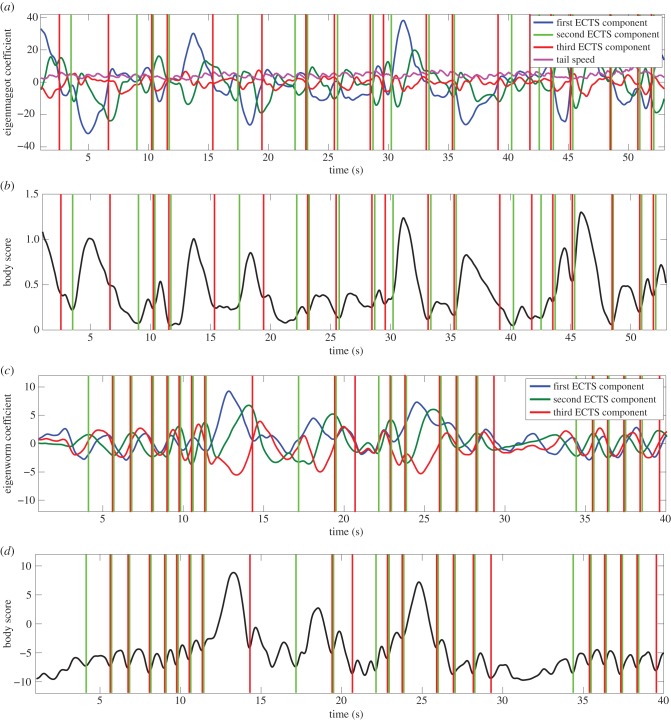
The segmentation algorithm. Panel (*a*) shows a screenshot of the larval ECTS and the tail speed time series. Panel (*b*) shows the corresponding body score, calculated as a weighted average of the ECTS dimensions, where the weights are set by the eigenvalue associated with each eigenshape. Local maximas and minimas in body score determine boundaries between actions, marked as green and red vertical lines for the beginning and end of actions respectively, in both panels (*a*) and (*b*). Panels (*c,d*) show the same information as (*a*,*b*) for *C. elegans*. The sinusoidal segments correspond to locomotion, note that segmentation resolves these into ‘steps'.

The intuition behind the segmentation algorithm is that boundaries between windows should be located where the dynamics of ECTS changes. ECTS was smoothed using a weighted running average filter with a window size of four frames and weights inversely proportional to the distance from the window's centre. Segmentation operates on a ‘body score’ time series that is created by calculating a weighted sum of the separate dimensions of ECTS, where the weights are set by the eigenvalues associated with the eigenshapes. The segmentation algorithm scans the body score to find local minima and maxima. An action is defined as a local maxima in body score bounded by minimas. The minimas define the start and end of the segmented subsequence. Figure 3 shows the result of segmentation for *Drosophila* and *C. elegans* with the corresponding body score time series.

The maxima/minima finding algorithm is controlled by a master parameter. The results are not strongly dependent on the precise parameter setting: adjusting it by ±25% leaves 92% of the annotation unchanged.

The behavioural videos of *C. elegans* were recorded while the worms were browsing in food. In this environment, worms often show low activity. Our segmentation was designed to identify periods where the body score rapidly changes, hence the identification of low activity periods required an extra step. Low activity periods were identified by intervals where the time derivative of body score remained under half of its average value for more than 0.5 s. These periods were added to the collection of actions prior to proceeding to the clustering step. If the two parameters (less than 50% of average body score for more than 0.5 s) are adjusted ±25%, then 97% of the action's classifications are not altered. Thus, fine tuning of the parameters is not necessary.

### 2.7. Curve alignment and clustering

Segmentation produces a large set of subsequences, or actions, each of which is a continuous ECTS curve. Hence, splines, locally smooth piecewise polynomials, are a natural choice to parametrize actions. Spline regression [12,13] was used to assign the actions to clusters. This method is analogous to Gaussian mixture models, but instead of Gaussian distributions, clusters are parametrized by splines.

To improve the consistency of spline fitting, the ECTS subsequences were aligned in the time domain. The frame with the highest body score was used as a reference, and actions were shifted in time such that their point of highest body score coincides, see electronic supplementary material, figure S2 for illustration. Note that if ECTS = [0, 0, 0], then the posture is a flat line (for both organisms). The higher the coefficients are, generally the more curved the postures are (although the bend caused by the coefficients can be in opposite directions and cancel each other). Therefore, the maxima of the body score correspond to the frame with the most bent posture and as such this frame is a rational choice to define a reference point in time by which subsequences of different lengths can be aligned.

Splines had three internal knot points and each polynomial had an order of 3. An expectation–maximization (EM) algorithm [13] was used to learn model parameters. EM was initiated 500 times with random boundary conditions, and the solution with the highest likelihood was kept. Bayesian information criteria (BIC) [18–20] was used to identify the optimal number of clusters. BIC is defined as2.1
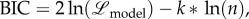
where 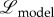 is the likelihood of the fitted model, *k* is the number of free parameters and *n* is the number of observations. The first term reflects goodness of fit of the model, and the second is a penalty term is for the number of free parameters.

Spline regression clustering produces a membership probability that a given action belongs to a cluster. Therefore, this method avoids rigid cluster assignments and also allows classification uncertainty to be quantified. To measure the classification uncertainty Shannon entropy [21] was used, defined as2.2
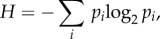


where *p_i_* is the probability that a given action belongs to a cluster *i*. Note that the most uncertain situation is when the probability is equally distributed among the clusters, correspondingly *H* has a maximum when all *p_i_* = 1/*i*_max_ (*i*_max_ is the number of clusters).

### 2.8. Comparison of behavioural annotations

In the following, a ‘behavioural event’ means an interval of consecutive frames tagged with the same behaviour. A behavioural event marked by an automated annotator (ESA, JAABA or CBD) was counted as true positive if at least 50% of it was also tagged by ground truth annotation with the same behaviour. Otherwise, the event was either counted as a false positive (automated annotator marked a behavioural event that had less than 50% overlap with an identically annotated behavioural event in the ground truth annotation) or a false negative (ground truth marked a behavioural event that had less than 50% overlap with an identically annotated behavioural event in the automated annotation).

Furthermore, we had to consider the problem that different annotations used different behavioural state spaces. The behaviours were always matched to the closest behaviour in the ground truth annotation. Specifically, for larval *Drosophila*, ESA's *turning manoeuvre* was treated as a match to both *stop cast* and *turn* in the ground truth annotation. That is, if ground truth contained either a *turn* or a *stop cast* behaviour and at least 50% of the frames were tagged as a *turning manoeuvre* by ESA, then it was counted as a true positive. *Run casts* are the same behaviour across ground truth, JAABA and ESA. For *C. elegans,* the ground truth hand annotation's *dwelling* was treated as a match to CBD's *pause* and ESA's *passive* state. The CBD's *ϒ* and *Ω* turns were both treated as a match to the ground truth's *turn* behaviour.

Parts of the time series were excluded from the analysis when the video frames could not be segmented and hence midline information was not accessible. Note that JAABA, CBD and ground truth annotation is available for these periods as they do not exclusively rely on contour information.

We modified the output of JAABA to avoid the problem of ‘flickering annotation’. Flickering annotation occurs when single frames within a behavioural event are not classified as part of the event, e.g. the sequence 0011011100 (where 1 means that the frame corresponds to a given behaviour, 0 means it does not). JAABA works on a frame-by-frame basis, hence these sequences are present when an event is near threshold value. To avoid the false positives caused by the small gaps, we have connected behavioural events that are less than three frames apart. Hence, the sequence above would become 0011111100.

To summarize annotation accuracy, we report the precision (positive predictive value) and sensitivity (also known as recall and true positive rate) [22] in tables 1 and 2. Sensitivity is the percentage of events recognized by the annotator, and precision is the proportion of events tagged by the annotator that are true positives. Furthermore, these two measures are combined as the *F*-score, defined as2.3
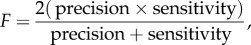
which is commonly used to quantify the goodness of classification.

**Table 1.** Statistics of the annotation of larval *Drosophila* behaviour. Precision, sensitivity and *F*-score values have been derived from electronic supplementary material, table S1. See electronic supplementary material, video S3 that shows the larva's behaviour ground truth, JAABA annotation and ESA annotation next to each other.

	run cast			stop cast			turn			all behaviours		
	Pre.	Sen.	F	Pre.	Sen.	F	Pre.	Sen.	F	Pre.	Sen.	F
JAABA	0.49	0.95	0.65	0.67	0.89	0.76	0.53	0.98	0.69	0.54	0.94	0.68
ESA	0.64	0.91	0.75	0.74	0.75	0.75	0.7	0.51	0.59	0.67	0.77	0.72

**Table 2.** Statistics of the annotation of *C. elegans* behaviour. Precision, sensitivity and *F*-score values have been derived from electronic supplementary material, table S2. See electronic supplementary, video S4 that shows the worm's behaviour ground truth, CBD annotation and ESA annotation next to each other.

	locomotion			turn			dwelling			all behaviours		
	Pre.	Sen.	F	Pre.	Sen.	F	Pre.	Sen.	F	Pre.	Sen.	F
CBD	0.77	1	0.87	0.96	0.79	0.87	0.89	0.94	0.92	0.86	0.9	0.88
ESA	0.83	0.93	0.9	0.67	1	0.8	0.73	0.83	0.77	0.74	0.95	0.82

### 2.9. Visualization, density cross sections and feature histograms

**Figure 4.**
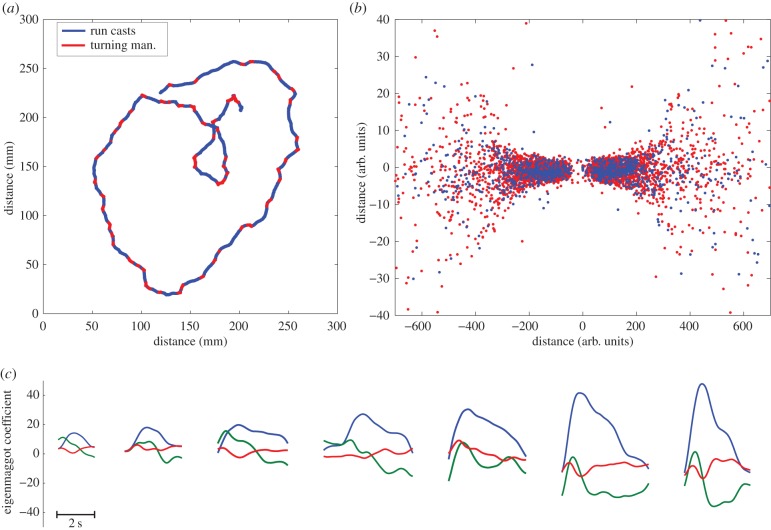
The structure of behavioural motifs for larval *Drosophila*. ESA identifies two motifs in the larva's behaviour; panel (*a*) shows a trajectory colour coded for the two motifs. Note that turning manoeuvres tend to happen when direction changes. Panel (*b*) shows a two-dimensional map of the distances among actions as measured by dynamic time warping (*R*^2^ = 0.85), see §2.9 for details. Panel (*b*) uses the same colour scheme as panel (*a*) to distinguish behaviours. The symmetry in the figure corresponds to the left/right symmetry in the animal's behaviour. Note that the points corresponding to the two behavioural motifs are concentrated in separate regions, yet there is no clear boundary between the two set of points. Panel (*c*) illustrates that similar ECTS subsequences can be found at every scale. These actions have been selected by starting in the middle of the map in (*b*) and picking example actions at regularly spaced distances along the *x*-axis, going from left to right.

**Figure 5.**
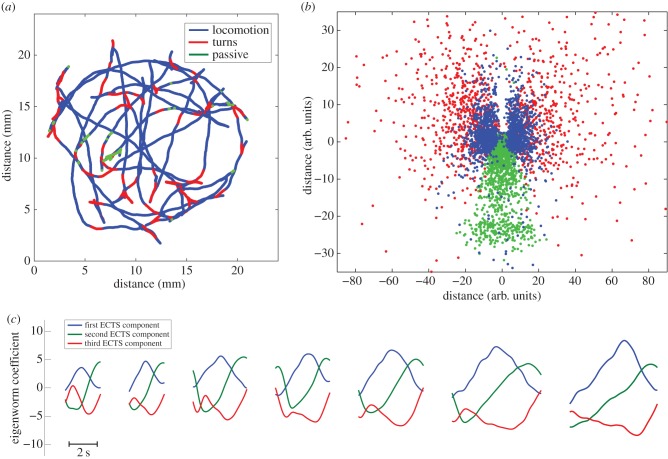
The structure of behavioural motifs for *C. elegans*. Panel (*a*) shows a trajectory colour coded for behaviour. Panel (*b*) shows a two-dimensional map of the distance among actions as measured by dynamic time warping (*R*^2^ = 0.78), see §2.9 for details. The symmetry in the figure corresponds to the dorsal/ventral symmetry in the animal's behaviour. Note that turn events are denser on the negative side of the *x*-axis. This effect is due to the ventral bias of *Ω*-turns [23]. Panel (*c*) illustrates that the ECTS subsequence corresponding to turns can be found at various scales, indicating that *Ω*-turns are not distinct behaviour, but a part of the continuum of turning behaviours.

To produce figures 4*b* and 5*b* and figure S1, the standard MATLAB [15] implementation of metric multidimensional scaling was used. The distance matrix was constructed using weighted dynamic time warping (DTW), where the weights are set by the eigenvalue associated with each dimension of ECTS. DTW is a standard measure of similarity in time-series analysis that uses a nonlinear time warping to find the optimal match between a pair of subsequences [24]. Note that the Euclidean distance among the points (corresponding to the actions) on the map correlates with the DTW distance among the subsequences, but the distances on the map are in arbitrary units. To construct each map, a random sample of 5000 actions were used. The algorithm was run 500 times with random initial conditions and the solution with the highest *R*^2^ was kept.

**Figure 6.**
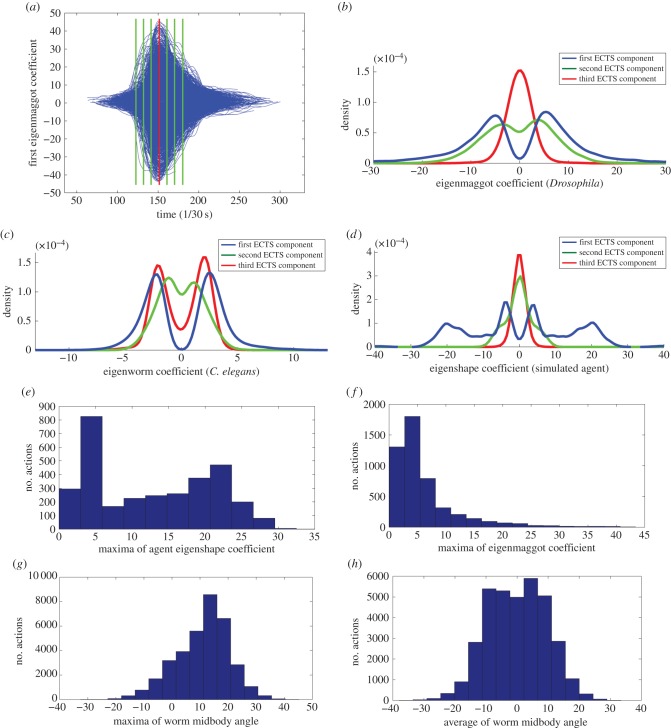
Continuity among behavioural states. Panel (*a*) shows the cross section taken across the aggregated ECTS curves. The cross section across the time of peak curvature (red line on *a*) is shown for *Drosophila* larva, *C. elegans* and the simulated agent on panel (*b–d*), respectively. For clarity, straight runs were removed from the agent's cross section. Panels (*e,f*) show the histogram of the maxima of first ECTS component during actions for the agent and *Drosophila* respectively. For the agent, the bimodal distribution indicates two distinct behaviours, but there is no clear cut-off amplitude for the real organism. Panel (*g,h*) shows the histogram of the maxima and the average of midbody bend for *C. elegans* actions. Again, we do not find a multimodal distributions, indicating that there is no data-defined threshold to distinguish separate behaviours.

The density cross sections of aggregated ECTS curves were visualized to see possible density fluctuations (see §3.4). Sets of stereotypical curves would form high-density regions in the cross sections. Hence, the cross sections can be used to detect stereotypical curves corresponding to stereotypical posture sequences. Density cross sections are measured on aggregated and aligned ECTS curves at specific ‘time slices' as shown in figure 6*a*. To estimate the density of curves, a kernel density estimation method was used [25]. Figure 6 only shows the cross section for one time slice, see electronic supplementary material, figure S3 for additional cross sections.

To create the histograms of *C. elegans* behavioural features, data were directly imported from the CBD feature files. These features are defined in [6]. The hardware and software that was used to obtain the behavioural features for larval *Drosophila* is described in [4].

## 3. Results

### 3.1. Eigenshapes

The eigenworm analysis pipeline extracts a vector of angles between consecutive points along the animal's midline, and applies principle component analysis to reduce the dimensionality of this description. The same method was adapted to create the analogous set of shapes for *Drosophila* larva, the eigenmaggots (figure 2). We find that eigenmaggots (figure 2*b*) are as efficient to describe larval postures as the eigenworms (figure 1*d*) are to describe worm postures. The inspection of eigenvalues reveals that three eigenmaggots account for over 90% of the postural variance [16] (figure 2*a*). Thus, eigenmaggots provide an accurate low-dimensional description of larval postures.

In contrast to eigenworms, eigenmaggots do not capture forward locomotion [7]. This difference is due to the different mode of locomotion. *C. elegans* propels itself by moving its body in a sinusoidal wave perpendicularly to the direction of motion [26]. Larval *Drosophila* crawls forward using peristaltic contraction waves [27]. The peristaltic waves can be recognized by the contraction of the abdominal sections, but this contraction does not alter the animal's midline shape from the camera's top view, and therefore is not captured by the eigenmaggot description. It is noted here that we have experimented with supplementing the larval ECTS with the tail speed time series as an extra dimension. The idea is that tail speed captures the state of peristalsis. However, the additional information did not improve the classification when evaluated against the ground truth annotation.

### 3.2. Motifs for *Drosophila* larva

For foraging *Drosophila* larva, the BIC for the spline regression model gave the best fit when assuming the presence of two behavioural motifs. The first motif we call a *run cast*. A run cast is a low amplitude head cast while the larva is moving approximately straight [28,29]. Successive run casts make up the larva's typical forward locomotion. The second motif corresponds to high amplitude head casts that may or may not be followed by a sharp change of direction. Some previous analyses of larval behaviour distinguish ‘stop casts' (or simply ‘casts'), where the larva stops locomotion and sweeps its head laterally, from ‘turns', which start in a bent body shape and end as the larva resumes locomotion in a new direction [4,9]. This classification scheme is not unique; others have proposed alternatives [30]. We do not find evidence to support the distinction between ‘stop casts' and ‘turns' instead our analysis describes these behaviours as a single motif, the *turning manoeuvre*. See electronic supplementary material, video S1 and figure 4 for an annotated trajectory and a visualization of the relationship among the motifs.

ESA annotation was evaluated against hand annotation. Across all behaviours, ESA produced an *F*-score of 0.72 (precision = 0.67 and sensitivity = 0.77), where the dominant source of error was a large number of false positive run casts. On the same behavioural experiments, JAABA annotation produced an *F*-score of 0.68 also with many false positive events. See table 1 for the precision, sensitivity and *F*-score statistics for each behaviour for both JAABA annotation and ESA. Electronic supplementary material, video S3 shows the binary video of the larva, hand annotation, JAABA and ESA annotations next to each other, so that the reader can gain a good understanding of how the different annotations relate to the larva's behaviour.

Typically, disagreements happen between ESA and hand annotation when an action has high classification uncertainty. Classification uncertainty is quantified by the Shannon entropy [21] and it is denoted by *H*. Seventy-three per cent of the ESA actions have a low uncertainty, meaning *H* < *H*_max_/4, where *H*_max_ = log_2_2, because two states have been found. For these low uncertainty actions, hand annotation and ESA agree on 87%. When classification entropy is high, *H* > *H*_max_/4, then the agreement rate between the two annotations drops to 49%. In short, action labels typically differ where ESA is uncertain. When hand annotation and ESA are in disagreement, it is often debatable which one is correct. In §3.4, we argue that the difficulty to resolve disagreements is due to an unbroken continuity between the two behavioural motifs.

### 3.3. Motifs for *Caenorhabditis elegans*

ESA was developed with the analysis of larval *Drosophila* in mind, but can also be applied to *C. elegans*. The worm behavioural data were obtained from the CBD. The database contains movies of worms browsing in bacteria, an environment where worms tend to pause for long periods. These pauses required an extra step in the segmentation process, see Methods for details.

In this case, BIC for the spline regression model fit indicated the presence of three behavioural motifs, corresponding to *locomotion*, *turns* and *passive periods*. Segmentation divides locomotion into ‘steps', where each step is a *π*/2 advancement of the locomotion wave. Multiple locomotion steps make up the characteristic undulatory motion of the worm. The turn behaviour as defined by ESA also includes classic *Ω* turns, lower amplitude turns and sharp pirouettes [23]. The passive periods are a mixture of pauses, dwelling and quiescence [31]. Figure 5 shows a visualization of the relationship between the motifs and an annotated trajectory, and electronic supplementary material, video S2 provides a dynamic illustration of the annotation.

To benchmark ESA, its performance was compared against hand annotation. ESA produced an *F*-score of 0.82 (precision = 0.74 and sensitivity = 0.95), where the dominant source of error was a large number of false positive turn events. This finding is not surprising given that the turning behaviour as defined by ESA is very permissive. Existing automated behavioural annotation of the CBD resulted in an *F*-score of 0.88 (precision = 0.86 and sensitivity = 0.9). See table 2 for the precision, sensitivity and *F*-score statistics for each behaviour for both CBD annotation and ESA. Furthermore, see electronic supplementary material, video S4, which shows the video of the worm, hand annotation, CBD and ESA annotations next to each other.

As for larval *Drosophila*, there is a significantly increased chance of a *C. elegans* action to be labelled differentially by ESA and hand annotation if the action has a high classification uncertainty (*H* > *H*_max_/4, where *H*_max_ = log_2_3 as three behavioural states have been detected) according to ESA. The probability that hand annotation labels these uncertain actions identically decreases to 39% from the population average 77%.

### 3.4. Do the larva and the worm exhibit discrete behaviours?

For both animals, the above analysis produces a substantial proportion of actions (around 25%) for which classification uncertainty is high. This suggests that the identified behaviours are not discrete, where ‘discrete’ means clearly distinguishable and stereotypical. Rather we see a continuous spectrum of behaviour. This is in contrast with the overwhelming majority of the literature that treats behaviour of these animals as a set of discrete states, although we are not the first to suggest a continuum among behavioural states for *C. elegans* [31].

To compare our results to what might be expected if there were discrete states, ESA was used to annotate the behaviour of an agent-based simulation of *Drosophila* larva which had been developed independently to study chemotaxis [32]. The agent's behaviour is controlled by a Markov chain model with three states: stop cast, run cast and straight run. Within each state, the precise motion (e.g. body bend) is determined by the current sensory conditions so can vary significantly. Videos were recorded of the agent in its virtual world, and the videos were put through the ESA pipeline (i.e. extracting eigenshape representation, segmentation, clustering). In this way, we test the ESA pipeline for its ability to detect underlying discrete states. We also present several alternative analyses that reveal distinct actions in the simulation but suggest a continuum of actions in the real animals.

#### 3.4.1. Clustering results

For the simulated agent ESA produced three clusters and for 94% of the time, it produced the same behavioural classification as ground truth annotation. BIC indicated a difference between the agent and the animals. For the agent, BIC provided strong evidence to distinguish the three clusters (ΔBIC_min_ = 7.57). In contrast, for both *Drosophila* larva and *C. elegans,* there was weak statistical evidence to justify the number of clusters (in both cases ΔBIC_min_ < 3.75) [33]. In other words, BIC is confident that there are three distinct clusters among the agent's actions, but for the two animals, the cluster structure is statistically much less justified.

#### 3.4.2. Structure in aggregated eigenshape coefficient time-series segments

We can directly examine this difference in cluster structure by visualizing the presence or absence of clear density bands in the aggregated ECTS subsequences (see §2.9). Sets of stereotypical curves form high-density regions in the cross sections, hence the cross sections can be used to detect stereotypical curves corresponding to a stereotypical posture sequences. Figure 6*a* shows the aggregated ECTS curves for the first ECTS component of larval *Drosophila*. Figure 6*b–d* shows the density cross sections for larval *Drosophila*, *C. elegans* and the agent, respectively. Note that the positive/negative asymmetry of ECTS values along the *x*-axis corresponds to the left/right asymmetry in larval behaviour and to the dorsal/ventral distinction for *C. elegans*. For both organisms, there is a single band in each half of the *x*-axis. This profile is in contrast with the two distinct bands of the agent's density cross section. The curves forming each high-density band correspond to one Markov state of the agent. Seven cross sections at various *x*-values were examined in each dimension for both the *C. elegans* and *Drosophila* (electronic supplementary material, figure S3), but they all had the same qualitative features as the cross section shown in figure 6, i.e. the animals do not have distinct bands that would support the inference of separable behavioural states.

#### 3.4.3. Structure in behavioural features

Weathervaning, or klinotaxis, is a steering process that results in the animal's trajectory bending towards higher concentration of odour [34]. For *Drosophila* larva, low amplitude head casts are hypothesized to be responsible for weathervaning [29]. These weathervaning casts are distinguished from head casts by the amplitude of body angle [28,29], which is very closely related to the amplitude of the first ECTS component, see figure 2*b*. The agent's behaviour was coded with this distinction in mind, so head casts tend to cause a higher body angle than weathervaning casts. Figure 6*e* shows the histogram of the maxima of first ECTS component during the agent's actions. The bimodal distribution clearly indicates two distinct behaviours. Based on this observation, we examined the maxima and average of a number of features of larval *Drosophila* (head speed, head angle, body angle, body angle speed and head angle speed) and *C. elegans* (eccentricity, head, midbody and tail angles) actions, see figure 6*e–h* and electronic supplementary material, figures S4 and S5. We hoped to find multimodal distributions and possibly sharp cut-off values because these could be used as data-defined thresholds to distinguish actions. However, in all cases, a smooth, unimodal distribution was found.

#### 3.4.4. Multidimensional scaling

A final way to examine this issue is to use multidimensional scaling to visualize the distance matrix of actions. DTW was used to measure distance, where the weights are set by the eigenvalue associated with each dimension of ECTS. Figures 4*b* and 5*b* show the larval *Drosophila* and *C. elegans* maps, respectively. As can be seen, there is no clear boundary in either figure to unambiguously separate behavioural motifs. This is in contrast with the agent's map, electronic supplementary material, figure S1, where clearly separated regions can be seen.

## 4. Discussion

This paper introduces eigenshape annotation, a bottom-up unsupervised method that searches for frequently repeated posture sequences in behavioural data. This problem is closely related to behavioural annotation, but not identical to it. Most behavioural annotators recognize behaviours through user-defined thresholds or training data [2–6]. In both cases, the set of possible behaviours and the description of those behaviours are determined by the user. In contrast, ESA is trying to discover the behavioural states directly from the data without any user input. Note that this task is considerably more challenging than behavioural annotation owing to the lack of *a priori* constraints. Thus, the novelty of this work is to create a data processing pipeline that discovers behavioural motifs in an unsupervised manner, where a behavioural motif is defined as a frequently repeated posture sequence.

The behavioural motifs discovered were generally consistent with behaviours described in the literature. However, many ESA motifs were more permissive than the definitions in other studies. For example, the ESA ‘turning manoeuvre’ for larva includes turns and high amplitude head casts [4], whereas the ESA ‘turning behaviour’ for the worm is a mixture of classic and wide *Ω* turns [6,23]. In both cases, there was no justification in the data for making any further subdivision of turns. Note that it can also be difficult for human observers to distinguish these behaviours consistently.

ESA was also unable to unambiguously classify many actions. The seeming continuity of the action distance maps, figures 4*b* and 5*b*, motivated us to further consider whether there are ‘defining features' that could objectively distinguish behaviours. In a simulated agent that was coded with distinct behavioural states, it is straightforward to find such features, for example, the amplitude of body bend (figure 6*e*). We searched for multimodal distributions in a variety of features of the *Drosophila* and *C. elegans* data, but failed in both cases. It remains possible that some feature we did not consider might reveal multimodality, or that discrete behaviours can be distinguished by considering a combination of multiple features.

There is an extensive literature that treats the behaviour of these animals as a set of discrete states. Despite our observation of continuity among behavioural states, our results are not necessarily in contradiction with the discrete treatment of behaviour. Discrete states can be seen as coarse graining (or binning) the continuous behavioural states. For example, the CBD defines *Ω* turns as a bend greater then *π*/6 propagating through the body. If the bend is between *π*/12 and *π*/6, then the event is called an *ϒ* turn. Thus, this classification scheme treats turning as a two state variable (*Ω*/*ϒ* turn). In contrast, ESA produces a membership probability that an action is a turn, instead of discretizing non-turns, *ϒ* and *Ω* turns at arbitrary thresholds. Coarse graining simplifies the underlying postural dynamics, and it can be an appropriate simplification for many studies. For example, the CBD's turn annotation is appropriate for studies looking at the worm's biased random walk. On the other hand, if an analysis requires the precise characterization of the worm's turning behaviour, then the continuous classification scheme of ESA can be advantageous.

However, adopting a coarse-grained description for convenience does not justify the widespread treatment in the research literature of behaviour as actually consisting of a set of discrete states, an assumption that needs to be independently evaluated. There is a risk that initially arbitrary distinctions between behaviours have become reified as qualitatively distinct behaviours of the animal, and treated as a set of actions between which it selects. For example, it is sometimes assumed that the underlying neural activity has a modularity that matches the behavioural states, and that this should guide investigation of neural circuits. In our results, the lack of stereotypical and distinguishable behavioural states suggests that the underlying neural activity is not stereotypical or modular. It remains possible that a highly stereotypical activity pattern of neurons implements a behavioural state, but owing to biomechanical effects, the resulting posture sequences are not so stereotypical. These alternate possibilities can only be addressed by studies of neural activity that do not exclusively depend on behavioural annotators that make *a priori* assumptions about the existence of discrete states.

A further possibility is that the lack of discrete actions observed in our study was a consequence of the particular behavioural conditions in which the animals were tested. Both environments were free of stimulus gradients: larval *Drosophila* was crawling on plain agar, whereas *C. elegans* was browsing in bacteria (although the bacterial layer could have minor inhomogeneities leading to shallow gradients). In future work, we will examine whether the behavioural space changes under different environmental conditions, for example, during directed chemotaxis in larval *Drosophila*.

ESA could be improved by advances in computer vision. Standard thresholding and skeletonizing algorithms fail when the animal intersects itself (2.5). The exclusion of self-intersecting postures introduces a bias to the pipeline, as no posture with self-intersection is included in the analysis. It is a possibility that there are discrete elements of behaviour in the self-intersecting sequences of postures.

The idea behind ESA is to find motifs in behaviour. We represented behaviour as posture, and posture as an ECTS, but the framework presented is not specific to either. ECTS can be replaced with any time series capturing behavioural features, or alternatively ECTS can be supplemented with such time series. Time series of higher-level features provide extra information for the classifier, potentially increasing its accuracy. For example, including a ‘direction of locomotion’ time series could lead to the detection of reversals as a separate state.

Alternative motif finding algorithms could be used on ECTS as well. For example, the subsequences yielded by segmentation can also be clustered using distance-based methods. We have experimented with several methods [35,36] in combination with standard distance measures (Euclidean and DTW), but it always led to results inferior to spline regression clustering in terms of the classification performance evaluated against hand annotation. We think that the performance difference is due to the ambiguous separation of clusters. Because of its probabilistic nature, spline regression clustering is better equipped to deal with datasets where many of the entries cannot be unambiguously classified.

Finally, we note that motif discovery is a challenging problem and it is an area of intense research in the machine learning community. Owing to the abundance of sequencing data most of the effort is focused on discrete, one-dimensional time series. To the best of our knowledge, the combination of segmentation and clustering is a novel approach to multidimensional motif finding. As discussed earlier, the framework is not specific to ECTS, therefore, we expect that with minor modifications the framework could also make contributions in other applications.

## Supplementary Material

ESM Figures, Tables, Video Captions

## Endnote

1 Code and data used for this publication are available at http://tinyurl.com/p7eculs.
