# Supplementary material for: Searching for motifs in the behaviour of larval Drosophila melanogaster and Caenorhabditis elegans reveals continuity between behavioural states
Source: J R Soc Interface. 2015 Dec 6;12(113):20150899. doi: 10.1098/rsif.2015.0899 (PMC4707863; doi:10.1098/rsif.2015.0899)
Supplement: ESM Figures, Tables, Video Captions [file rsif20150899supp1.pdf]

Supplementary figures

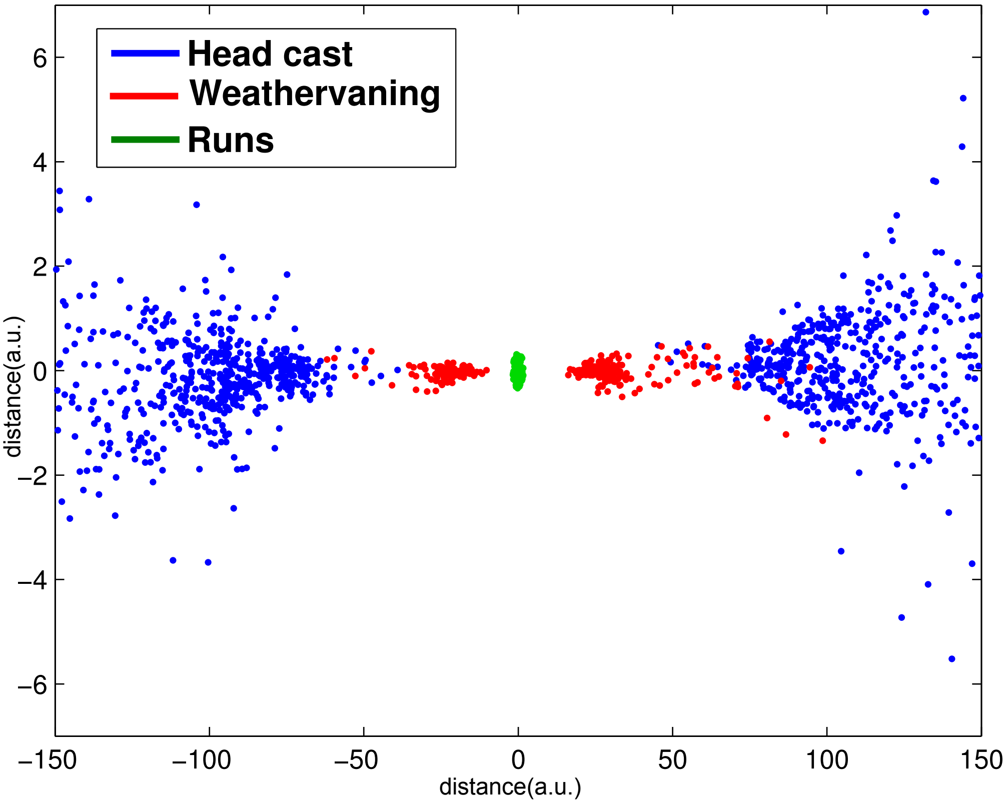

Figure S1: Multidimensional scaling visualisation of the agent's actions ($R^2 = 0.74$), see the *Methods* for details. Note that unlike for the two organisms there is a distinct boundary separating behavioural motifs. This separation is due to the agent behaviour being driven by a Markov model that consists of discrete states.

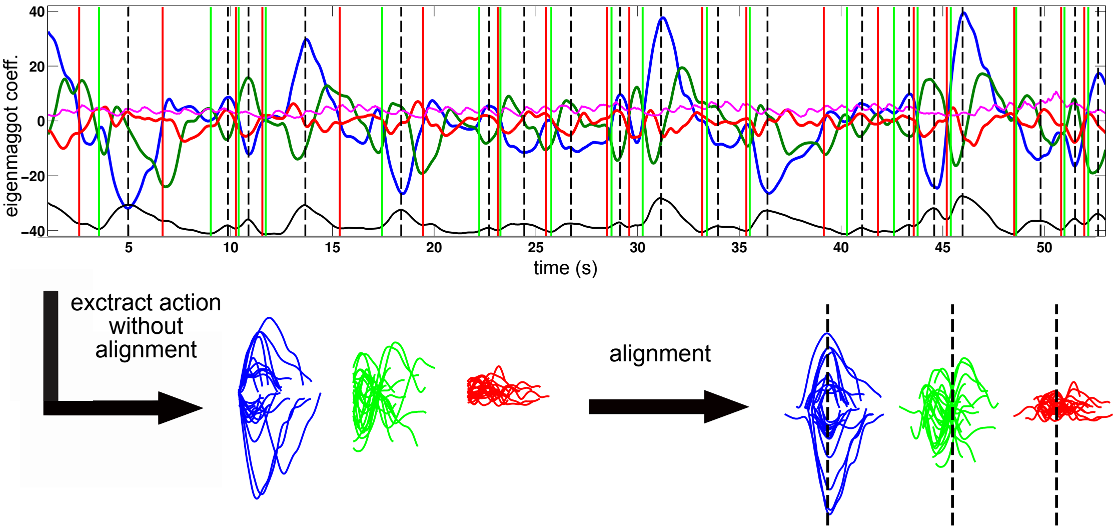

Figure S2: Alignment of actions. Figure shows a window of *Drosophila* behaviour, with the ECTS components and the body score at the bottom. For each action the frame with the highest BS value (dashed black line) was used as a reference. Actions were shifted in time such that their point of highest BS coincidences. The frame with the highest body score defines the most curved posture during actions, therefore it provides a rational choice to define the midpoint of actions (in time). Every data analysis was done on time-aligned actions.

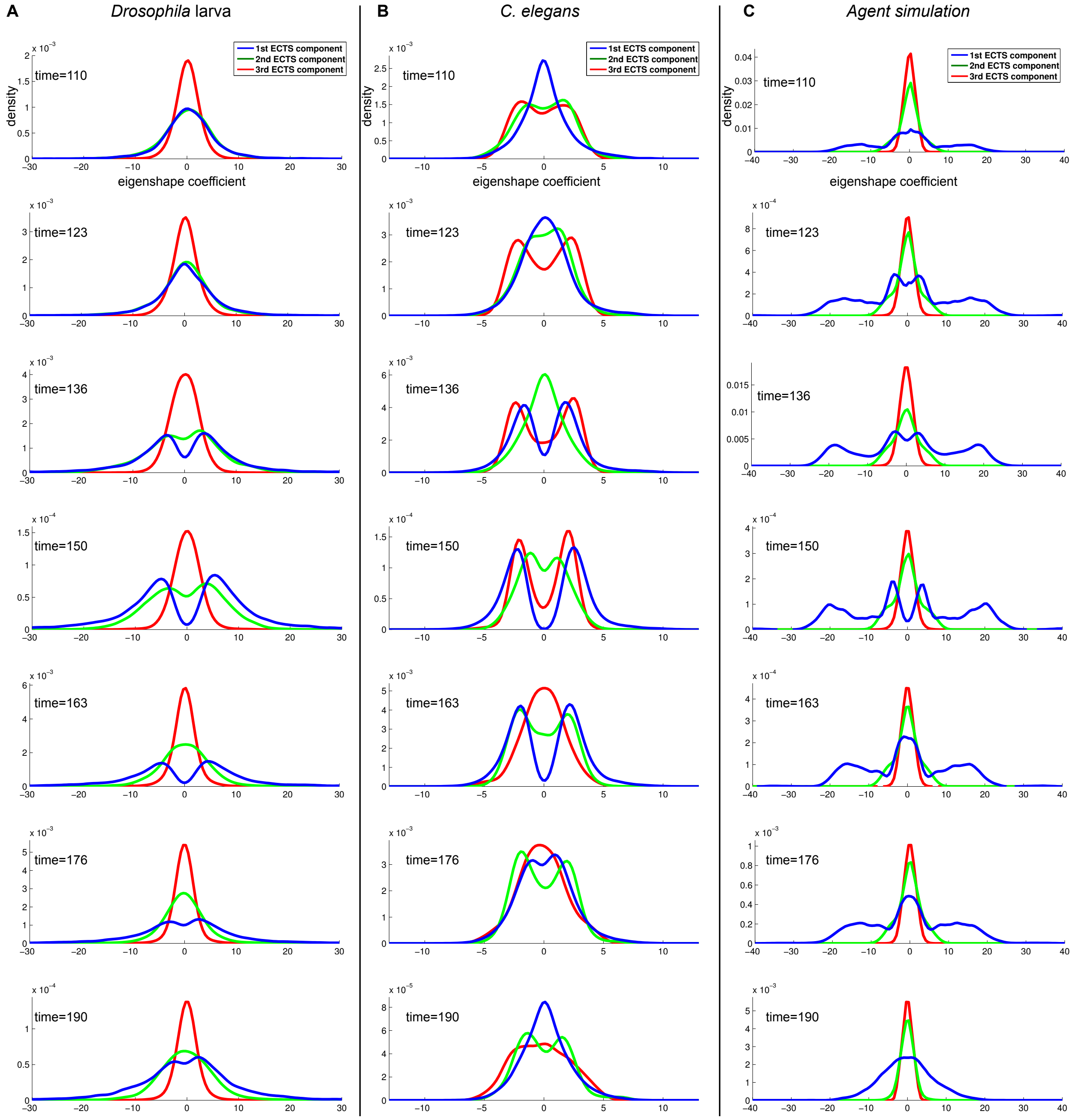

Figure S3: Density cross sections of aggregated ECTS curves. Each column shows density cross sections for larval *Drosophila*, *C. elegans* and the agent simulation. Each row corresponds to a different time slice, see Figure 5A for explanation. Note that in this case time is given in frame numbers, that is each increment in t corresponds to 1/30s. Bands in this figure correspond to typical ECTS curves, which represent typical sequences of body shapes. With the exception of the agent simulation, each curve is smooth on each side of 0, indicating a lack of well defined discrete states.

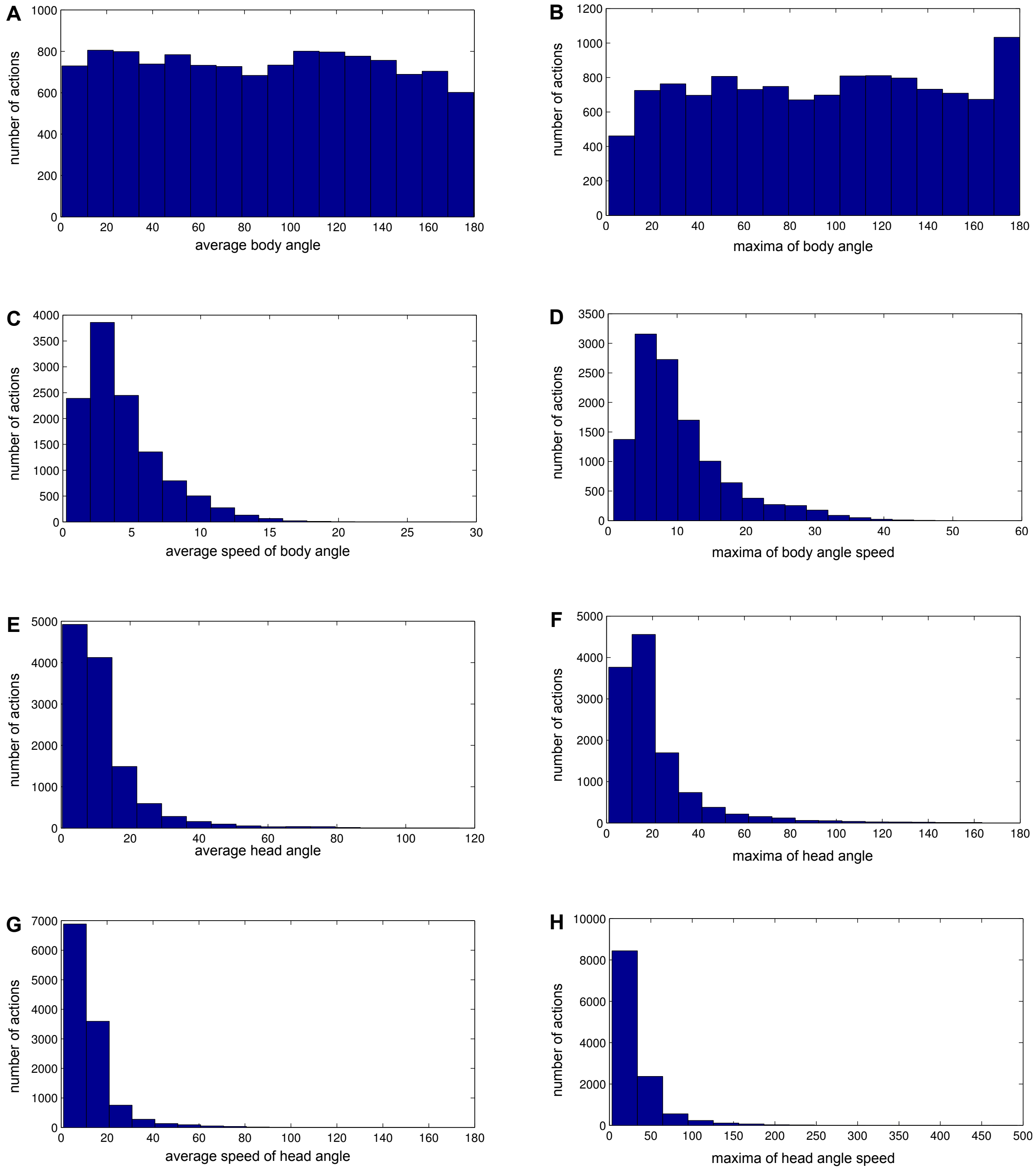

Figure S4: Histograms of larval *Drosophila* action features. In the following both the maxima and the averages are measured on individual actions, that is each action contributes a single data point to these histograms. **A** and **B** displays the histogram of average and maxima of body angles, while **C** and **D** shows the histogram of average and maxima of the speed body angle change. Similarly **E** and **F** depicts the histogram of average and maxima of head angles, while **G** and **H** shows the histogram of average and maxima of the speed head angle change. For a precise definition of these features see [4].

There is little evidence for multimodality in these distributions.

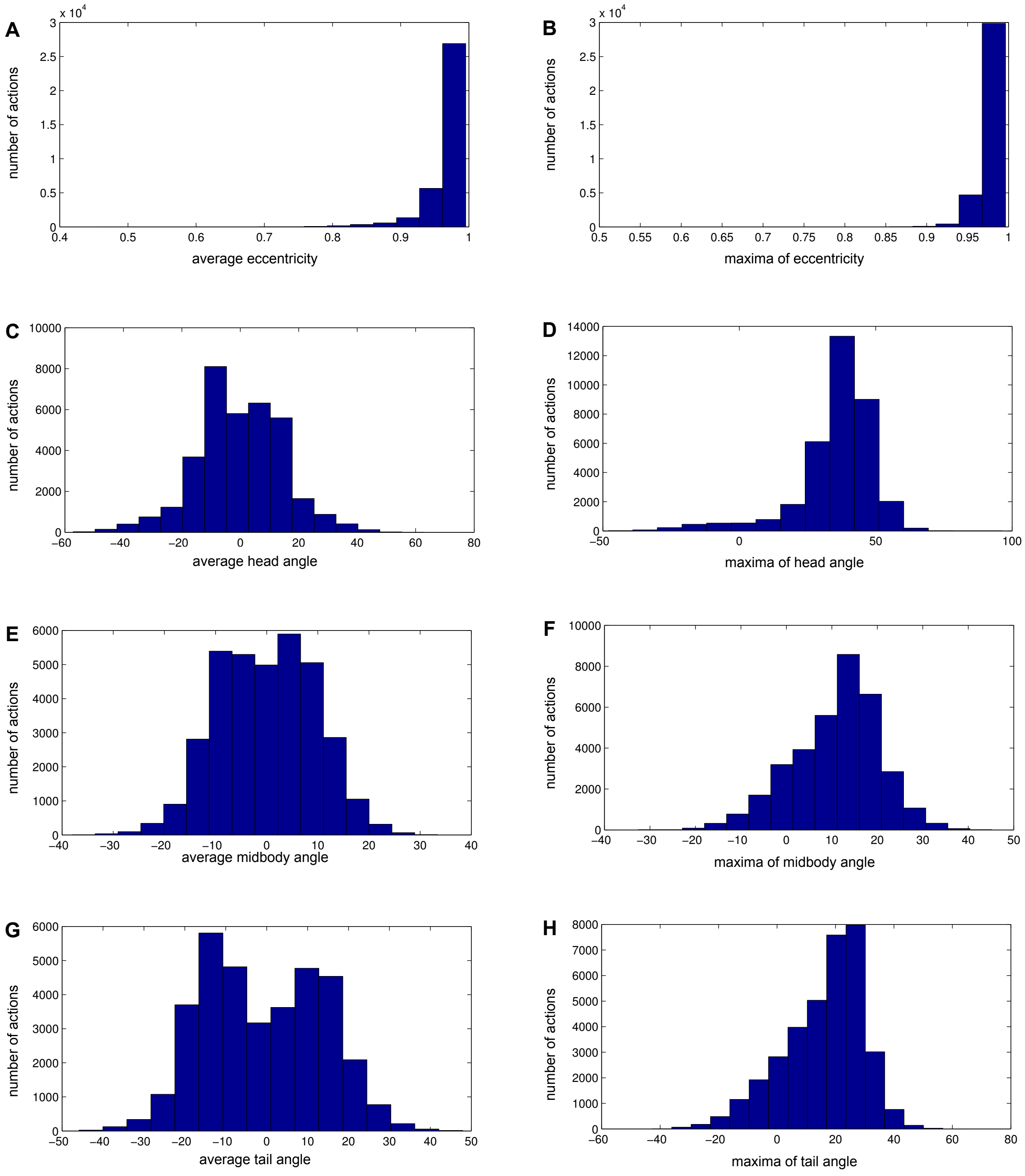

Figure S5: Histograms of *C. elegans* action features. In the following both the maxima and the averages are measured on individual actions, that is each action contributes a single data point to these histograms. **A** and **B** displays the histogram of average and maxima of eccentricity. **C** and **D** shows the histogram of average and maxima of head angle. Similarly **E** and **F** depicts the histogram of average and maxima of mid body angles, note that the asymmetry along the x-axis corresponds to the dorsal/ventral asymmetry in behaviour. Panels **G** and **H** show the histogram of average and maxima of tail angle. For a precise definition of these features see [6].

Supplementary tables

	Run Cast			Stop Cast			Turn			All behaviours		
	TP	FP	FN	TP	FP	FN	TP	FP	FN	TP	FP	FN
JAABA	114	117	6	54	27	7	44	39	1	212	183	14
ESA	109	61	11	34	12	11	31	13	30	174	86	52

Table S1: Numerical comparison of the annotation of larval *Drosophila* behaviour. TP, FP and FN stands for true positive, false positive and false negative respectively. See *Methods* for a detailed description how the matches were counted.

	Locomotion			Turn			Dwelling			All behaviours		
	TP	FP	FN	TP	FP	FN	TP	FP	FN	TP	FP	FN
CBD	66	20	0	55	2	15	34	4	2	155	26	17
ESA	65	13	1	70	34	0	29	12	7	164	59	8

Table S2: Numerical comparison of the annotation of *C. elegans* behaviour. TP, FP and FN stands for true positive, false positive and false negative respectively. See *Methods* for a detailed description how the matches were counted.

Video captions

SV1(http://tinyurl.com/nuhmubp - maggotESAannotation.mp4) Segmentation of larval ECTS. Top left panel shows a binary video of a tracking experiment. Top right panel shows the midline of the larva reconstructed from ECTS. The middle panel show the time evolution of the first three components of ECTS. Green and red vertical lines mark the beginning and the end of actions respectively. Note that if the end of an action coincidences with the beginning of the next action, then only red vertical line will show. The blue vertical line in the middle marks the time corresponding to the video frame in the two top panels. Bottom panel shows the behavioural annotation of ESA. The annotation is probabilistic, height corresponds to the probability that the action is an example of a certain behaviour.

SV2 (http://tinyurl.com/onx3ph2 - wormESAannotation.mp4) Segmentation of worm ECTS. Top left panel shows video of a tracking experiment with the midline and contour of the worm highlighted. Data was acquired from the *C. elegans* behavioural database. Top right panel shows the midline of the worm reconstructed from ECTS. The middle panel show the time evolution of the first three components of ECTS. Green and red vertical lines mark the beginning and end of actions respectively. Note that if the end of an action coincidences with the beginning of the next action, then only red vertical line will show. The blue vertical line in the middle marks the time corresponding to the video frame in the two top panels. Bottom panel shows the behavioural annotation of ESA. The annotation is probabilistic, height corresponds to the probability that the action is an example of a certain behaviour.

SV3 (http://tinyurl.com/pkxzqar - maggotAnnotationCompare.mp4) Comparison of larval behavioural annotations. Top left panel shows a binary video of a tracking experiment, while the top right panel shows the midline of the larva reconstructed from ECTS. The bottom panel shows the ground truth, JAABA and ESA annotation going from top to bottom. For better visualization all three annotations are shown on the same panel, but height only has significance for the ESA annotation. ESA annotation is probabilistic, height corresponds to the probability that the action is an example of a certain behaviour. For ground truth and JAABA, the annotation is binary (either 1 or 0). The blue vertical

line in the middle marks the time corresponding to the video frame in the two top panels. When midline disappears in the top right panel, the vision algorithm could not isolate the larva from the background. For small gaps (less then 15 frames) ECTS was interpolated. Wider gaps could not be reliable interpolated, hence these times were excluded from the ESA analysis.

SV4 (http://tinyurl.com/nace2lc - wormAnnotationCompare.mp4) Comparison of worm behavioural annotations. Top left panel shows a video of a tracking experiment, while the top right panel shows the midline of the larva reconstructed from ECTS. The bottom panel shows the ground truth, CBD and ESA annotation going from top to bottom. For better visualization all three annotations are shown on the same panel, but height only has significance for the ESA annotation. ESA annotation is probabilistic, height corresponds to the probability that the action is an example of a certain behaviour. For ground truth and CBD, the annotation is binary (either 1 or 0). The blue vertical line in the middle marks the time corresponding to the video frame in the two top panels. When midline disappears in the top right panel, the vision algorithm could not isolate the larva from the background. For small gaps (less then 15 frames) ECTS was interpolated. Wider gaps could not be reliable interpolated, hence these times were excluded from the ESA analysis.
